# Detection, characterization, and enrollment of donors of Ebola convalescent plasma in Sierra Leone

**DOI:** 10.1111/trf.14580

**Published:** 2018-03-23

**Authors:** Richard S. Tedder, Dhan Samuel, Steve Dicks, Janet T. Scott, Samreen Ijaz, Catherine C. Smith, Charlene Adaken, Christine Cole, Samuel Baker, Tansy Edwards, Philip Kamara, Osman Kargbo, Saidia Niazi, Davis Nwakanma, Umberto d'Alessandro, Graham Burch, Heidi Doughty, Colin S. Brown, Nick Andrews, Judith R. Glynn, Johan van Griensven, Ebola_CP Consortium Investigators, Georgios Pollakis, William A. Paxton, Malcolm G. Semple

**Affiliations:** ^1^ Blood Borne Virus Unit; ^2^ Serology Development Unit, Virus Reference Department National Infection Service, Public Health England; ^3^ Transfusion Microbiology National Health Service Blood and Transplant; ^4^ Division of Infection and Immunity University College London London UK; ^5^ Institute of Translational Medicine; ^6^ Institute of Infection and Global Health National Institute for Health Research (NIHR) Health Protection Research Unit in Emerging and Zoonotic Infections, University of Liverpool Liverpool UK; ^7^ Travel Medicine and International Health Team Health Protection Scotland Glasgow UK; ^8^ Clinical RM Ohio USA & Connaught Hospital; ^9^ National Safe Blood Service, Connaught Hospital, Ministry of Health and Sanitation Freetown Sierra Leone; ^10^ Department of Infectious Disease Epidemiology London School of Hygiene and Tropical Medicine; ^11^ Department of Disease Control, Faculty of Infectious and Tropical Diseases London School of Hygiene and Tropical Medicine London UK; ^12^ Medical Research Council, Fajara Banjul The Gambia; ^13^ DiaSorin S.p.A, Biotechnology Manufacturing Dartford UK; ^14^ National Health Service Blood and Transplant; ^15^ College of Medical and Dental Sciences University of Birmingham Birmingham UK; ^16^ King's Sierra Leone Partnership, King's Centre for Global Health, King's Health Partners and King's College London; ^17^ Reference Microbiology, National Infection Service; ^18^ Statistics, Modelling and Economics Department Public Health England London UK; ^19^ Department of Clinical Sciences Institute of Tropical Medicine Antwerp Belgium

## Abstract

**BACKGROUND:**

Passive therapy with convalescent plasma provides an early opportunity to intervene in Ebola virus disease (EVD). Methods for field screening and selection of potential donors and quantifying plasma antibody are needed.

**STUDY DESIGN AND METHODS:**

Recombinant Ebola virus glycoprotein (EBOV GP) was formatted into immunoglobulin G‐capture, competitive, and double‐antigen bridging enzyme immunoassays (EIAs). EVD survivors in Freetown, Sierra Leone, were recruited as potential plasma donors and assessed locally using sera alone and/or paired sera and oral fluids (ORFs). Uninfected controls comprised unexposed Gambians and communities in Western Area, Sierra Leone. Antibody neutralization in selected sera was measured retrospectively in a pseudotype virus assay.

**RESULTS:**

A total of 115 potential donors were considered for enrollment: 110 plasma samples were concordantly reactive in the three EIAs; three were concordantly unreactive and two were reactive in two of three EIAs (98.2% agreement; 95% confidence interval [CI], 93.9%‐99.8%). In 88 donors with paired ORF and plasma, G‐capture EIA reactivity correlated well in the two analytes (R^2^ = 0.795). Plasma and ORF from 44 Gambians were unreactive. ORF samples from 338 of 339 unexposed Western Area community controls were unreactive (specificity, 99.7%; 95% CI, 98.4%‐99.7%); ORF samples from 113 of 116 Kerry Town EVD survivors were reactive (sensitivity, 97.4%; 95% CI, 92.5%‐99.5%). Strong reactivity in G‐capture and/or competitive EIAs identified donors with high plasma EBOV GP antibody levels in the double‐antigen bridging assay, correlating with high levels of neutralizing antibody.

**CONCLUSIONS:**

In‐field testing can qualify convalescent donors for providing high‐titer antibody.

**ABBREVIATIONS**
CP: convalescent plasma
DABA: double‐antigen bridging assay
EBOV: Ebola virus
ETU: Ebola treatment unit
EVD: Ebola virus disease
GMT: geometric mean titer
GP: glycoprotein
IC_50_: 50% reduction of virus infection
NOD: normalized optical density
ORF(s): oral fluid(s)
TM: transport medium

By late 2014 the outbreak of Ebola virus disease (EVD) was unchecked in Guinea, Liberia, and Sierra Leone. The potential efficacy of convalescent plasma (CP) as a treatment for EVD, first described in the 1995 Kikwit outbreak,^1^, ^2^, ^3^ led the World Health Organization (WHO) to consider development of CP therapy a priority.^4^, ^5^ CP has been used for other viral infections^3^ and we have previously shown^6^ the importance of serologic confirmation of potential plasma donors rather than relying on a syndromic diagnosis alone.

The antibody response after clinical EVD appears long‐lived^7^ and correlates with the presence of neutralizing antibody. Nonhuman primate studies have shown that antibody to EBOV envelope glycoprotein (EBOV GP) protected against disease progression. *A priori*, and supported by these studies in nonhuman primates,^9^ we decided to ensure that human CP was drawn only from donors with plasma levels of EBOV GP antibody likely also to contain high levels of neutralizing antibody. To deliver this aspiration new serologic methods for the detection and qualification of immune donors were needed.^10^ The development of assays was undertaken, including for noninvasive samples, to provide initial screening as well as the identification of those seropositive potential donors with higher antibody levels. In spite of previous attempts to use nonblood analytes including oral fluid (ORF) for Ebola serology being unsuccessful,^11^ recognizing the widespread use of ORF in clinical virology,^12^ we also elected to pursue this approach. The enzyme immunoassays (EIAs) had to be specific and semiquantitative, preferably including noninvasive protocols, and sufficiently robust to be transported and used in the field in the absence of a cold chain. A series of EIA formats were constructed using the recombinant EBOV GP antigen employed by Qiu and colleagues.^9^ The use of these EIAs, including a noninvasive test using ORF, for facilitating the identification, recruitment, and qualification of convalescent blood donors as a potential source of therapeutic CP is described.

## MATERIALS AND METHODS

### Potential donors

The study “Convalescent plasma for early Ebola virus disease in Sierra Leone” (ISRCTN13990511 and PACTR201602001355272) was approved by the Sierra Leone Ethics and Scientific Review Committee, authorized by Pharmacy Board of Sierra Leone (PBSL/CTAN/MOHS‐CST001) and sponsored by the University of Liverpool. The protocol is available on request (m.g.semple@liverpool.ac.uk).

**Figure 1:**
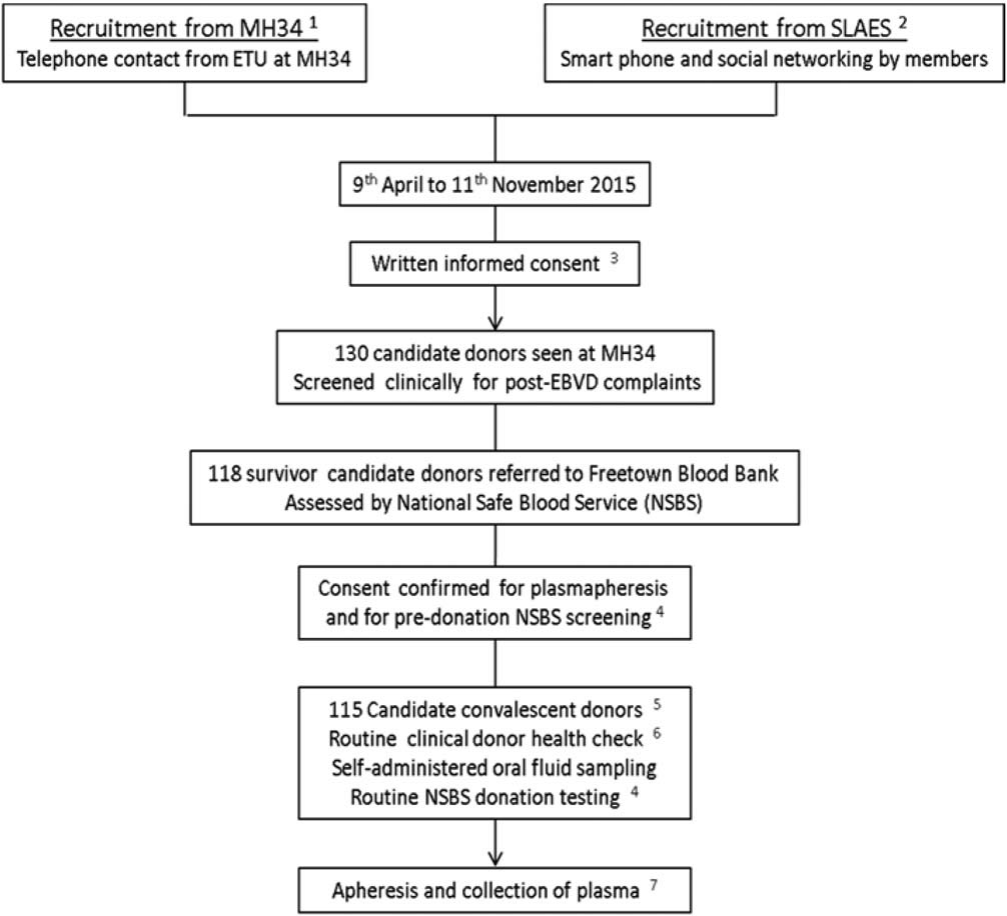
Recruitment process for volunteer convalescent donors seen first at the 34th Regimental Military Hospital Wilberforce Freetown (MH34) before referral to the Blood Bank, National Safe Blood Service, Connaught Hospital, Freetown. ^1^34th Regiment Military Hospital, Wilberforce, Freetown, Sierra Leone. ^2^Sierra Leone Association of Ebola Survivors (SLAES). ^3^Compensation for cost of attendance of 40,000 Sierra Leone Leones (SLL, $8 USD). ^4^Anemia, HBsAg, anti‐HCV, anti‐HIV, and antibody to syphilis. ^5^HBV infection excluded three donors; compensation of 80,000SLL ($16 USD) for attendance. ^6^Current well‐being; vital signs including temperature, height, and weight; research samples including ORF sampling by Oracol device; blood drawn in tempus tubes, vacutainer PPT tubes, and EDTA tubes. ^7^Candidate donors returned to NSBS Blood Bank for first and subsequent apheresis and received compensation for each apheresis of 300,000 SLL ($60 USD).

Ebola virus disease survivors with certificates (issued by Ebola Treatment Centers on discharge) were recruited as potential donors through Military Hospital 34, Freetown, and the Sierra Leone Association of Ebola Survivors. Of 130 recruited and consented (Fig. 1), on average 6.1 months (range, 1‐15 months; midquartiles, 4‐8 months) after discharge, 12 were referred to the Ebola Survivors' Clinic because of possible post EVD sequelae.^13^ A total of 118 well volunteers were referred to the blood bank in Freetown for final assessment. Three were excluded because of hepatitis B virus infection. The remaining 115 provided written consent to become qualified donors, giving 315 plasma samples and 205 ORF samples overall. In addition the first (#15/220) and the second (#15/252) WHO standards from the National Institute of Biological Standards and Controls were included as controls.

### Unexposed controls, The Gambia

Forty‐four individuals were enrolled by the Medical Research Council Unit, The Gambia, into a malaria field study and serum and ORF samples archived. Approval was given to test these locally for antibody to EBOV.

### Survivors and unexposed community controls, Sierra Leone

As part of a separate study on transmission within households^14^ ORF samples were taken from 116 polymerase chain reaction (PCR)‐confirmed EBV survivors from Kerry Town Ebola Treatment Center, and 339 healthy volunteers living in three communities in Western Area Sierra Leone, which had no recorded cases of EVD. These samples were analyzed in Makeni, Sierra Leone. This study was approved by the Sierra Leone Ethics and Scientific Review Committee and by the ethics committee of the London School of Hygiene and Tropical Medicine. Written consent was sought from all participants or their parents or guardians if under 18 years.

### ORF sampling

The (Oracol (S10, Malvern Medical Developments Limited) ORF sampling kit) was used. In Connaught blood bank the oral swabs were demonstrated by blood bank staff and then self‐administered by donors. The swab was run along the upper and lower gum margins on either side of the mouth for 30 seconds each (2 min total) before returning it to the sampling tube. Samples were tested on receipt or stored at –20°C within a day of collection.

For the Kerry Town survivors and the community controls the oral swabs were demonstrated by the field staff and then self‐administered, with adults helping children. Each swab was rubbed firmly on the gums for 90 seconds, sealed, put in a cool box, transferred daily to the laboratory, and tested on receipt or stored at –20°C as above.

### Sample handling

Whole blood samples from potential donors were separated within 24 hours of venesection. All plasma samples received in Public Health England Colindale were tested for EBOV RNA.^15^ Fresh ORFs or frozen ORFs that had been thawed at room temperature were extracted by adding 1 mL of transport medium (TM) to the tube, agitating the swab in the TM within the tube, removing the swab with a circular motion, dispelling ORF and TM from the swab to provide the ORF extract, which remained in the tube. Plasma samples and ORF extracts were stored at 4°C for up to 24 hours to allow testing before long‐term storage at –20°C.

### EIAs

Three solid‐phase microplate EIAs were formulated, based on the EBOV Mayinga GP antigen (rGPδTM, IBT Bioservices, Inc., USA Cat. 0501‐016). In brief, the first was an immunoglobulin (Ig)G‐specific reverse capture assay (G‐capture EIA) using horseradish peroxidase (HRP)‐labeled EBOV GP. The second was a simultaneous competitive assay (competitive EIA) using HRP‐labeled monoclonal antibody (MoAb; 4G7) raised against EBOV GP. The third was a double‐antigen bridging assay (DABA) using HRP‐EBOV GP performed qualitatively or quantitatively to measure antibody to EBOV GP (full details of assays, critical reagents, and information for use leaflets in the Supplementary Information, available as supporting information in the online version of this paper). Both latter assays would be expected variously to detect IgM antibody, the competitive less so because of expected antibody low avidity in acute infection, the DABA more so because of the pentameric nature of IgM.

The positive control for the first two kits was plasma from a UK EVD survivor infected in Sierra Leone taken 1.5 months after discharge. The DABA EIA used a pool of five reactive donor plasmas attributed 1000 arbitrary units/mL (au/mL). The negative control plasma came from unexposed UK blood donors.

### Normalized optical density measures

Optical densities (ODs) were “normalized” as the ratio between the test sample and the cut‐off. For G‐capture and DABA, where a reaction is defined by a test sample giving an OD of at least the kit cutoff, the normalized optical density (NOD) value is derived by test sample OD/cutoff OD.

For the competitive EIA, where a reaction is defined by a test sample giving an OD of not more than the kit cutoff, the NOD value is derived by cutoff OD/test sample OD. Samples giving NOD of at least 1.0 are reactive.

### Neutralizing antibody

The ability of a sample to neutralize the propagation of a single‐cycle infectious EBOV GP pseudo‐type, as previously described,^16^ was used to investigate a selected panel of CP samples (details in Supplementary Information). In brief, the envelope‐deficient human immunodeficiency virus (HIV)‐1 backbone pSG3^Δenv^ from the NIH AIDS reagents repository (https://www.aidsreagents.org/) was complemented with the Ebola GP expressed in (pcDNA3.1 from Thermofisher). The Ebola GP is derived from the KP096421 early strain to which the coding changes appearing later in the epidemic (A81V, I317V, T229A, and N551D) were introduced. All analyses were performed in triplicate and repeated. The neutralizing ability of a sample was expressed as that dilution of plasma that provided a 50% reduction of virus infection (IC_50_).

### Sample size and statistical analysis

The size of the potential donor panel was determined by the need for plasma for a planned therapeutic intervention study. Within‐ and between‐assay variability was assessed as the percentage of coefficient of variation (CV; i.e., standard deviation/mean) using paired testing of samples and from repeat testing of the positive kit control across runs. Results from different assays (DABA, G‐capture, competitive) run on the same samples as well as G‐capture run on paired plasma and ORF samples were compared by calculating R^2^ values (square of the correlation coefficient) and as percentage agreement.

## RESULTS

### Test performance

For the G‐capture EIA, replicate testing within plates of 222 analytes (plasma or ORF) from the potential donors, gave a mean CV of 2.9%; replicate testing of ORF samples from survivors and community controls gave an average CV of 8.0% within plates (23 samples) and 17.9% between plates (104 samples). For the competitive EIA, replicate testing within plates of 127 plasma samples from potential donors gave a mean CV of 9.1%. For the quantitative DABA on repeat testing the mean CV was 13%, the calibration plots demonstrated a CV of approximately 8%.

### Control populations

Forty‐four ORF and serum samples were tested in duplicate at Medical Research Council, The Gambia. With a cutoff of kit mean negative plus 0.1, no sample was considered reactive. The 16 serum samples with highest OD reactions were retested in the competitive EIA; all were unreactive.

In Sierra Leone, using the same cutoff of mean negative plus 0.1 OD, ORF from 338 of 339 individuals with no known exposure to EBOV infection were unreactive, giving a specificity of 99.7% (95% confidence interval [CI], 98.4%‐99.9%). The one reactive sample (NOD = 1.4) was unreactive on further duplicate retesting. All other samples had NOD of less than 0.7. Among the 116 PCR‐confirmed survivors from Kerry Town Ebola Treatment Center, 113 ORF samples were reactive on a single test, giving a sensitivity of 97.4%, (95% CI, 92.5%‐99.5%).

### Potential donors

**Figure 2:**
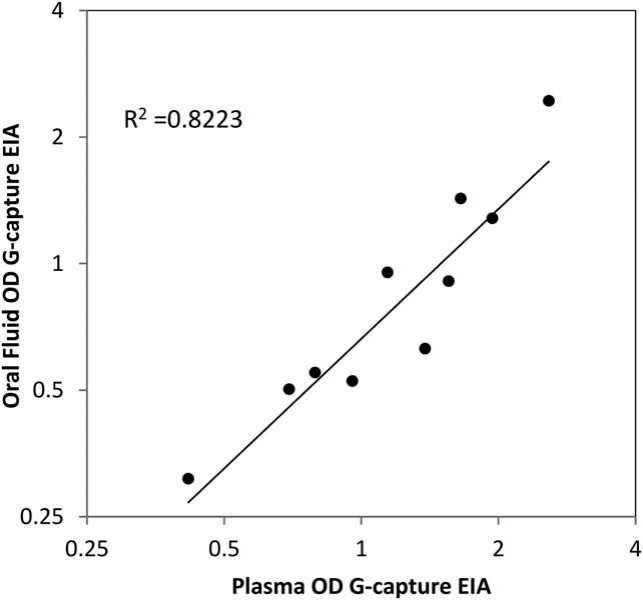
Correlation between paired ORF and plasma reactivity, expressed as raw OD, from 10 convalescent donors tested in the G‐capture EIA at Connaught Blood Bank, Freetown (R^2^ = 0.822 from linear regression). Linear regression line on logged titers is shown.

**Figure 3:**
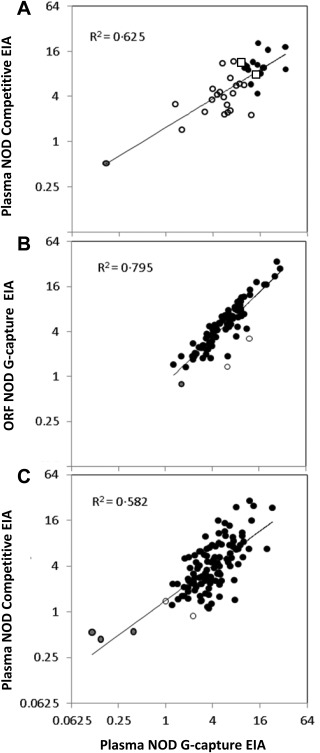
(A) Correlation of NOD in the competitive and the G‐capture EIAs of 37 donor samples field tested in Connaught Blood Bank, Freetown (R^2^ = 0.625 from linear regression). (●) Samples from donors selected for further attendance. One sample is concordantly unreactive (●). (◻) Reactivities of the two WHO standards (15/262 top left; 15/220 bottom right). (B) Correlation between NOD reactivity of 88 paired ORF and plasma samples in the G‐capture EIA from donors taken at first attendance (R^2^ = 0.795). One ORF sample had a NOD value less than 1.0 (●, 0.78). (○) Two dually reactive plasmas with anomalously low ORF NODs. Linear regression line on logged titers is shown. (C) Correlation between G‐capture and competitive EIAs, expressed as log NOD values, of 115 first‐attendance plasma samples (R^2^ = 0.582). Three samples are concordantly unreactive in both EIAs (●). Two samples are discordantly unreactive (○), one is just below the cutoff in the G‐capture, and the other is just below cut‐off in the Competitive EIA (see Table 1). Linear regression line on logged titers is shown.

Field testing of paired plasma and ORF samples from 10 convalescent donors tested using the G‐capture EIA at Connaught Blood Bank in May 2015 demonstrated a clear correlation between the reactivity of plasma and ORF (Fig. 2; R^2^ = 0.822). Tested at Connaught at the same time, 36 of 37 convalescent donor plasma samples, were reactive in both G‐capture and competitive EIAs, with correlated reactivity levels (Fig. 3A; R^2^= 0.625). Further initial and repeat retesting was carried out at Public Health England Colindale. All 115 plasma samples were retested for EBOV RNA on receipt in the UK; all were negative. Eighty‐eight paired plasma and ORF samples showed good correlation in reactivity on the G‐capture EIA (R^2^ =0.795 using linear regression; Fig. 3B). One ORF sample was unreactive (NOD, 0.78), giving a sensitivity of ORF compared to plasma of 87 of 88, 98.9% (95% CI, 93.8%‐99.97%). Two ORF samples, although reactive, had lower reactivity in the ORF G‐capture test than expected from the plasma results (Fig. 3B). They had a low level of IgG in the ORF as sampled (<3 mg/L). One of these donors (ORF NOD, 3.2; plasma NOD, 12.0) had provided an unpaired ORF 3 days earlier which gave a NOD of 10.0.

Plasma samples from the 115 donors were tested in the G‐capture, competitive, and DABA EIAs: 110 were concordantly positive in all three EIAs, three were concordantly unreactive in all three EIAs, and two samples were below the cutoff in one of the three EIAs but reactive in the other two (Table 1). The overall agreement between the three assays was therefore 113 of 115 (98.2%; 95% CI, 93.9%‐99.8%) and the sensitivity of the assays was 111 of 115 (96.5%; 95% CI, 91.3%‐99.0%) for G‐capture and competitive EIAs and 112 of 115 (97.4%; 95% CI, 92.6%‐99.4%) for DABA. Reactivity correlated between competitive and G‐capture EIAs (Fig. 3C).

**Table 1:** Details of the five first‐time donor plasma samplings where an EIA NOD was less than 1.0 in one or more of the three EIAs^a^

Sample identity	G‐capture EIA		Competitive EIA		DABA EIA (au/mL)	Comment
	Raw OD	NOD	Raw OD	NOD		
Donor 1	0.02	0.15	3.20	0.43	<35^b^	PCR cycle threshold 20 in holding unit, undetectable at 48 and 72 hr later when retested after transfer to ETU.
Donor 2	0.02	0.11	2.58	0.54	<35^b^	No record of PCR found nationally for this donor by name within 4 days of the date of reported admission to an ETU.
Donor 3	0.07	0.38	2.53	0.55	<35^b^	No record of PCR found nationally for this donor by name within 4 days of the date of reported admission to an ETU .
Donor 4	0.17	0.99	1.00	1.38	112	Recorded PCR positive (though discrepancy in sex and age in records) no address provided.
Donor 5	0.39	2.24	1.57	0.88	64	PCR‐positive cycle thresholds 34 and 37 in two tests taken a day apart.
Positive control	3.65	20.87	0.14	18.26	1000	UK 1 plasma used for both G‐capture and competitive EIAs. A pool of highly reactive plasma ascribed to contain 1000 au used for DABA.
Negative control	0.07	0.41	2.63	0.53	<35^b^	Pooled normal human plasma from UK blood donors.
Cutoff	0.17	1.00	1.39	1.00	Not applicable	Defined for G‐capture by mean OD‐negative controls + 0.1 OD. Defined for competitive EIA by comparison with 50% inhibition of label binding.

a Plasma samples from Donors 1‐3 inclusive were unreactive in any of the three tests used. Plasma sample from Donor 4 was unreactive in the G‐capture EIA and plasma sample from Donor 5 was unreactive in the competitive EIA, both plasmas from Donors 4 and 5 contained detectable antibody to EBOV GP in the DABA EIA.

b Lower limit of detection in the run.

**Figure 4:**
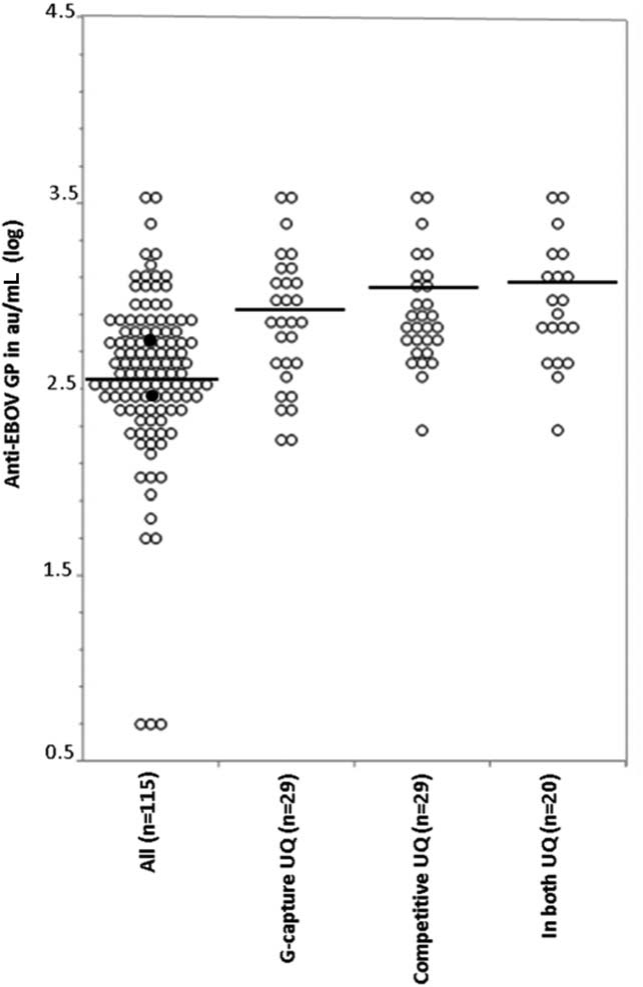
Anti‐EBOV GP levels in 115 seropositive convalescent donor plasmas, expressed as log_10_ au/mL, measured in the DABA EIA. Results are shown for the entire cohort (All) superimposed with the first (15/220, lower of the two) and second (15/262, upper of the two) WHO EBOV standards (●) and for those in either of the top quadrants for the G‐capture (Capture UQ) or for the competitive EIA (Competitive UQ) and for those plasma samples reacting in both top quadrants of the G‐capture and the competitive EIAs (In both UQ). Horizontal bars represent geometric mean values anti‐EBOV GP in au/mL.

**Figure 5:**
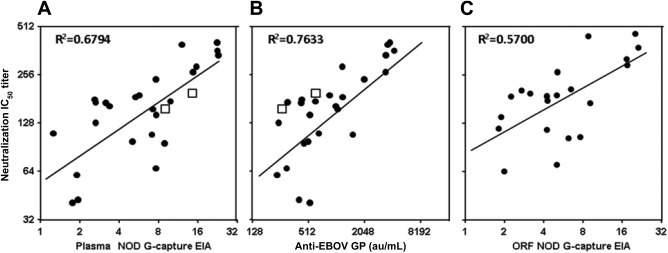
Anti‐EBOV GP levels in a selected panel of 25 convalescent donors. Plasma antibody measured by pseudotype neutralization (interpolated IC_50_ neutralization titers) correlates with plasma reactivity in the G‐capture EIA (expressed as NODs, A, R^2^ = 0.6794), with quantified plasma reactivity in the DABA EIA (expressed in au/mL, B, R^2^= 0.7633) and with paired ORF (21 ORF samples only) reactivity in G‐capture EIA (expressed as NODs, C, R^2^ = 0.5700). (◻) IC_50_ titers (A, B) for first (15/220 bottom left) and the second (15/252 bottom right) WHO standards at 162 and 192, respectively. Linear regression lines on logged titers are shown.

EBOV GP antibody reactivity was measured in the quantitative DABA EIA (Fig. 4). The three samples concordantly unreactive in the two screening EIAs, had undetectable EBOV GP antibody in the qualitative DABA EIA. The measurable antibody in the remaining 112 plasma samples varied over 2 × log, from 50 to 3624 au/mL with a geometric mean titer (GMT) of 392 au/mL. The 29 samples with reactivity in the upper quartile of the capture EIA had GMT 745 au/mL, the 29 in the upper quartile of the competitive EIA had a GMT of 838 au/mL, and the 20 in both upper quartiles had a GMT of 934 au/mL. The subset of 25 samples chosen for investigation of neutralizing antibody had DABA antibody levels ranging from 200 to more 4000 au/mL reflecting the range of reactivity of donor samples in this assay. There was a close correlation between DABA reactivity and IC_50_ titers of neutralizing antibody (R^2^ = 0.7633, Fig. 5B). A similar correlation between the level of neutralizing antibody and reactivity in the G‐capture EIA was also seen for the paired plasmas (R^2^ = 0.6794, Fig. 5A) and ORFs from 21 of the same donor samplings where ORF data were available (R^2^ = 0.5700, Fig. 5C). The neutralization IC_50_ of both the first (#79 NIBSC 15/220) and the second (#92 NIBSC 15/262) WHO standards are as shown at 162 and 192, respectively (Figs. 5A and 5B).

## DISCUSSION

We have shown that recruitment and screening of potential CP donors to exclude seronegative individuals and to select those with higher antibody levels is possible in a resource‐poor setting during an EVD outbreak. Furthermore, the high sensitivity and specificity of the tests developed and the comparable performance of the G‐capture EIA when used on ORFs have implications beyond the identification of donors, as they enable large‐scale noninvasive serologic studies.^14^

Seronegative individuals were, however, rare among potential donors (3/115). Although all potential donors possessed certificates indicating discharge from an Ebola treatment unit (ETU), in the societal turmoil of an ongoing epidemic and resulting deprivation it is not surprising that certification may not be secure. Although recruitment was facilitated by certification and financial incentive, it is clearly appropriate to use serology to qualify donors for therapeutic purposes. The seronegative donor of Plasma Sample 1 (Table 1) was diagnosed before admission to the ETU with a high viral load, which became undetectable within 48 hours suggesting an erroneous first PCR procedure. On further inquiry, no record of EBOV PCR testing could be found nationally for the seronegative donors of Plasma Samples 2 and 3. If these three are truly seronegative as these data would infer, the G‐capture EIA identified 111 of 112 (99.1%; 95% CI, 95.1%‐99.98%).

Oral fluid testing provides acceptable noninvasive sampling, used widely in clinical virology^12^ and is valuable in acute outbreaks and seroepidemiologic studies. The ORF EIA also correlated with EBOV candidate vaccine response in UK volunteers.^17^ The negative G‐capture results from a nonexposed population in The Gambia indicated good specificity. This was confirmed in the unexposed community controls from Sierra Leone (338/339 negative; specificity, 99.7%) while the sensitivity of the G‐capture EIA on ORF remained high (113/116 Kerry Town EVD survivors positive; sensitivity, 97.4%).^14^ Furthermore, the clear association between reactivity of ORF and plasma (Figs. 2 and 3B) demonstrates that ORF is appropriate for investigating the spread of EBOV in diverse communities as well as selection of seropositive donors. Previous studies were unable to detect EBOV antibody in ORF samples^11^ reflecting the poor sensitivity of indirect EIAs for ORF studies.^18^ This is not the case for reverse G‐capture EIAs. However, the ORF sample must be taken adequately to avoid false negatives from low‐ORF IgG levels; it would not normally be possible in the field to check total IgG levels in the ORF.

A competitive EIA incorporating a MoAb to a well‐defined neutralizing epitope^9^ confirmed the specificity of the ORF G‐capture EIA, but requires a plasma sample. The use of EIAs of different format has long been considered advantageous in the terms of specificity^19^ and this same principle should apply to EBOV serology. Choice of the EBOV GP antigen for serology was driven by availability, by the previous selection of this GP for vaccine studies and the generation of protective murine MoAb.^9^ This, however, does not imply that antibody to EBOV GP is necessarily the only therapeutic component of CP.

Having an antigen‐coated solid phase and a directly conjugated GP, it was a natural extension to develop a double‐antigen bridging EIA for antibody quantification. Measurement of the level of reactivity in a capture or competitive assay has subtly different implications. A capture assay reaction depends on the proportion of the antibody present in the analyte that recognizes the antigen and the avidity with which the antibody interaction occurs. A competitive assay depends on the concentration of antibody present in the analyte and the avidity and specificity of that antibody. Usually a strong reaction in one EIA correlates with a strong reaction in the other EIA, but not necessarily with a direct linear relationship (Figs. 3A and 3C). When antibody to EBOV GP was quantified in the DABA EIA, levels differed widely between individuals and many survivors had very low levels of measurable antibody to GP. This suggests that alternative host determinants such as the cytotoxic T‐lymphocyte response may be more important for survival and recovery than the humoral response.^8^ It also begs the question whether EVD survivors with low antibody levels are more susceptible to viral persistence or reactivation.

Both the G‐capture and the competitive EIAs allowed selection of donors with high‐level antibody (Fig. 4) quantified by DABA EIA, which in turn measures total antibody to EBOV GP. This selection, however, reduced the number of suitable donors available, so having a noninvasive initial screening method that could be used more widely was considered particularly useful. The range of antibody levels we observed across the cohort may explain the lack of clinical benefit^4^ found with the use of unselected CP in the trial in Guinea. Quantification of antibody to EBOV GP in DABA correlated with the measurement of biologically determined neutralizing antibody. Both assays also ranked the two WHO standards in the expected order of potency and the observed neutralization IC_50_ titers were in agreement with published data.^20^ We believe that these observations indicate that the DABA EIA was suitable for quantifying biologically active antibody in the field. If the effect of CP depends on antibody dosing,^10^ it will be interesting to quantify neutralizing antibody in the full cohort of Sierra Leone donors.

The three different EIAs we have developed, their biologic plausibility, correlation with neutralizing antibody, and the excellent performance of the G‐capture EIA on ORF providing a sensitive, specific, and noninvasive way of determining the EBOV serologic status of individuals, provide a suitable epitaph to our much loved and sadly missed colleague Dr Dhan Samuel.

## Supporting information

Additional Supporting Information may be found in the online version of this article at the publisher's website.

Supporting Information

## APPENDIX A

### Ebola_CP Consortium Investigators

The Ebola_CP: The Consortium Investigators for Ebola_CP (Convalescent Plasma for Early Ebola Virus Disease in Sierra Leone) are M.G. Semple (Consortium Lead Investigator) and J.T. Scott, (both Institute of Translational Medicine and NIHR Health Protection Research Unit in Emerging and Zoonotic Infections University of Liverpool, Liverpool, UK); S.M. Gevao (Country Lead Investigator), F. Sahr (Country Deputy Lead Investigator), C.P. Cole and J. Russell (all College of Medicine and Allied Health Sciences, Freetown, Sierra Leone); S. Baker, O. Kargbo, and P. Kamara (all National Safe Blood Service, Connaught Hospital, Ministry of Health & Sanitation, Freetown, Sierra Leone); M. Lado and C.S. Brown (King's Sierra Leone Health Partnership, King's Health Partners & King's College London, London, UK); J. van Griensven, R. Ravinetto, and Y. Claeys (all Institute of Tropical Medicine, Antwerp, Belgium); R.S. Tedder, R. Gopal, and T.J.G. Brooks (National Infection Service, Public Health England, London, UK); C.C. Smith (Health Protection Scotland, UK); H.A. Doughty (NHS Blood and Transplant & College of Medical and Dental Sciences, University of Birmingham, Birmingham, UK); A. Mari Saez and M. Borchert (both Institute for Tropical Medicine and International Health, Charité, Berlin, Germany); A.H. Kelly (Department of Sociology, Philosophy and Anthropology, Exeter University, Exeter, UK); J.K. Baillie (The Roslin Institute, University of Edinburgh, Edinburgh, UK); N. Shindo, and D. Pfeifer (Department of Pandemic and Epidemic Diseases, World Health Organization, Geneva, Switzerland); D.L. Hoover (ClinicalRM Inc., Hinckley, OH); W.A. Fischer II and D.A. Wohl (both Department of Medicine, University of North Carolina, Chapel Hill, NC); N.M. Thielman (Duke University School of Medicine, Durham, NC); P.W. Horby and L. Merson (Nuffield Department of Medicine, University of Oxford, Oxford, UK); and P.G. Smith and T. Edwards (Medical Research Council Tropical Epidemiology Group, London School of Hygiene & Tropical Medicine, London, UK).

### CONFLICT OF INTEREST

The authors have disclosed no conflicts of interest.
