# Supplementary material for: Detection, characterization, and enrollment of donors of Ebola convalescent plasma in Sierra Leone
Source: Transfusion. 2018 Mar 23;58(5):1289–98. doi: 10.1111/trf.14580 (PMC5947131; doi:10.1111/trf.14580)
Supplement: Supplementary file 1 — Supporting Information [file TRF-58-1289-s001.pdf]

Supplementary Information for Methods
Transfusion

Section	Page
Critical reagents for EBOV antibody kits	2
IFU Leaflet: Double Antigen Bridging Assay (DABA) for the Quantification of antibody to Ebola Virus Glycoprotein	3-14
IFU Leaflet: EBOLA DABA Qualitative Assay	15
IFU Leaflet: Competitive enzyme-linked immunosorbent assay for the detection of antibody to ebola virus in human serum or plasma	16-19
IFU Leaflet: Immunoglobulin G capture enzyme immunoassay for the detection of antibodies to Ebola virus in human serum, plasma or oral fluid	20-23
Measurement of neutralising antibody by pseudo-type inhibition	24

Critical reagents for EBOV antibody kits

Antibody solid phase

100 µl of polyclonal rabbit anti-sera to human ɣ-Fc (Jackson Immuno Research Cat: 309-005-008) at 5 microgrammes/ml were used to coat microtitre wells , washed and then quenched by the addition of 200 µl of MicroImmune Blocking Buffer (Cat: 20011) and stabilised by drying overnight at 37° C.

Antigen solid phases

EBOV GP Mayinga strain (Integrated Bio Therapeutics Bioservices, Gaithersburg, US; ΔTM EBOV GP; Cat: 0501-016). 100 µl of antigen (0·5 microgrammes/ml for the competitive assay or 2 microgrammes/ml for the DABA) in Coating Buffer (Clin Tech, Guildford, Cat: 643003) at pH 7·6 were used to coat microtitre wells (Nunc Immunomodule, U8 Maxisorp) over night at 4°C , followed by 3 hours at 37°C. After washing the solid phase was quenched by the addition of 200 µl of MicroImmune Blocking Buffer (Cat: 20011) and stabilised by drying overnight at 37° C.

Buffers

Conjugate diluent, sample diluent, Washing buffer (Glycine Borate Buffer) and stop solution (2M H_2SO_4) were gifted by DiaSorin UK (Dartford, UK).

Conjugates

ΔTM EBOV GP conjugated to horseradish peroxidase (HRPO) using a succinimidyl protocol stored in a stabilising solution (Clin Tech, Guildford Cat: PN20080) at 4°C.

Murine monoclonal antibody 4G7 (gifted by Gary Kobinger, Winnipeg, Canada) conjugated and stored as above.

Substrate

Ready to Use TMB substrate (MicroImmune Ltd Cat: 20030b)

Transport medium (TM)

Phosphate-buffered saline, pH 7·2-7·4, supplemented with 10% v/v Foetal Bovine Serum, 0·1% v/v tween-20, 0·05%v/v red dye and antibiotics Amphotericin B 0·5µg/ml and Gentamicin 0·25mg/ml.

Double Antigen Bridging Assay (DABA) for the Quantification of Antibody to Ebola Virus Glycoprotein

1. SUMMARY

EBOV Double Antigen Bridging Assay (DABA) is developed to detect and quantify anti-EBOV antibodies to Zaire ebolavirus (as an arbitrary unit). EBOV DABA has been developed and designed to be extremely sensitive and specific using recombinant proteins: recombinant ZEBOV GP (IBT Bioservices) and recombinant ZEBOV GP-HRP.

2. INTENDED USE

This kit is an enzyme-linked immunosorbent assay for the quantitative detection of antibodies to Zaire ebolavirus in human serum or plasma. It is intended as a confirmatory assay for individuals who were screen reactive using the EBOV G-capture assay and provide an indication of plasma antibody titre based upon an arbitrary unit.

3. PRINCIPLE OF THE TEST

DABA Enzyme Linked Immunoassays utilise a recombinant or purified antigen pre-coated onto a ‘solid phase’ (usually polystyrene microwell strips), which acts as a capturer. A second antigen conjugated to tracer enzyme-horseradish peroxidase (HRP) is used as the detector. In the presence of EBOV antibodies, the pre-coated and conjugated antigens will be bound to the two variable domains of the antibody during incubation, thus a specific antigen-antibody-antigen-HRP immuno complex is developed and captured on the solid phase. After the addition of TMB Substrate, the colourless chromogens are hydrolysed by the bound HRP conjugate to a blue coloured product. The blue colour turns yellow after stopping the reaction with 2M sulphuric acid. The presence of colour within the test well indicates the presence of antibodies to EBOV in the sample. The amount of colour can be measured and is proportional to the amount of antibody present. Wells containing samples negative for EBOV antibody remain colourless.

4. REAGENTS

Each kit contains sufficient materials for 96 tests. The shelf life of each kit is as indicated on the label fixed to the box containing the kit. All components must be stored at 2-8°C unless otherwise stated.

Materials Provided:

**Coated Wells**:	1 plate of 96 wells coated with recombinant ZEBOV GP. Allow the wells to reach room temperature (18-30°C) before removal from the bag. Place unused wells in the sealable storage bag provided and return to 2-8°C.
**Negative Control**:	One bottle containing 200 µl of Negative Control. Ready to use.
**Standard controls**	1x set of nine standards containing 60ul of each standard control. Ready to use. **NB: vortex/centrifuge each tube to ensure the full volume of standard control is in the bottom in the tube prior to opening.**<br>**The standard set equates to the following Arbitrary Units (au) per ml;**<br>**Standard 1 = 1000au/ml**<br>**Standard 2 = 500au/ml**<br>**Standard 3 = 250au/ml**<br>**Standard 4 = 125au/ml**<br>**Standard 5 = 62.5au/ml**<br>**Standard 6 = 31.25/ml**<br>**Standard 7 = 15.63au/ml**<br>**Standard 8 = 7.81au/ml**<br>**Standard 9 = 3.91au/ml**
**Sample Diluent:**	One bottle containing 16 ml of Sample Diluent
**Conjugate Diluent:**	One bottle containing 10 ml of Conjugate Diluent
**Conjugate Concentrate:**	One bottle containing 100 µl of 100 times strength recombinant EBOV Zaire glycoprotein coupled with horseradish peroxidase antigen. **NB: vortex/centrifuge tube to ensure the full volume of conjugate concentrate is in the bottom in the**

	**tube prior to opening.**<br><br>Add one volume of Conjugate Concentrate to 99 volumes of Conjugate Diluent to get the required volume of Conjugate Solution.
**TMB Substrate – Ready to use: (Supplied by Microimmune Ltd)**	One bottle containing 10 ml of 3,3',5,5'- tetramethylbenzidine (TMB) and stabilisers in a colourless solution (TMB substrate – ready to use)<br><br>Keep substrate away from sunlight. The Substrate Solution should be colourless; if it is purple before being used, it should be discarded and fresh Substrate Solution used.<br><br>Once opened the bottle of TMB substrate is stable refrigerated (2-8°C) for 30 days, but must be discarded if crystals have formed.
**Stop Solution (2M H_2SO_4)**:	One bottle containing 5 ml of 2M sulphuric acid (H_2SO_4) ready to use.
**Wash Fluid**:	One bottle containing 125 ml of 20 times working strength of Glycine Borate Wash Solution.<br><br>Add one volume of Wash Fluid Concentrate to 19 volumes of distilled or deionised water to give the required volume or dilute the entire contents of one bottle of Wash Fluid to a final volume of 2500 ml.<br><br>Store the working strength wash fluid at 18-30°C in a closed vessel under which conditions it will retain activity for one month

Additional Material and Instruments Required But Not Provided:

Good quality deionised or distilled water
Clean vessels for wash solution preparation
Microtitre plate cover
Micropipettes and disposable tips capable of delivering 200 µL, 100 µL, 20 µL and 1-5µl volumes.
Waste discard container with disinfectant
EIA plate reader capable of reading optical density at 450nm (and 620-650nm).
Incubator, 37°C

5. SPECIMEN COLLECTION AND PREPARTION

Handle all blood, serum and plasma as potentially infectious material.
Serum and plasma (EDTA, citrated or heparinised) samples are suitable specimens for the test and should be obtained from whole blood using standard laboratory procedure.

If not used immediately, they can be stored at 2-8°C for one week. Care should be taken to ensure that the serum samples are clear and not contaminated by microorganisms. Plasma samples collected into EDTA, sodium citrate or heparin may be tested, but highly lipaemic, icteric or haemolysed samples should be used as they can give false results in the assay. Do not heat inactive samples.

6. STORAGE AND STABILITY

When stored at 2-8°C, the kit is stable up to the expiration date printed on the kit label.

7. PRECAUTIONS AND SAFETY

The standard Controls and Negative Controls are not reactive for antibodies to HIV 1 and 2, HCV and for Hepatitis B surface antigen. The controls should be handled and disposed of as though potentially infectious.

The TMB substrate solution contains 3, 3', 5, 5'-tetramethylbenzidine and has been reported to be non-carcinogenic. Avoid direct skin- contact with the reagent. Wear latex gloves when handling this reagent. If TMB comes into contact with the skin, wash immediately with water.

The Stop Solution contains sulphuric acid (2M). Contact with skin and mucous membranes should be avoided. If the Stop Solution comes into contact with these sites, rinse with copious amounts of water.

Wear disposable gloves when handling clinical specimens and kit components. Treat all clinical specimens and controls and any materials coming into contact with them as potentially infectious.

Dispose clinical material and potentially infected materials in accordance with local regulations.

Avoid microbial contamination of reagents. Do not use reagents that show signs of contamination.

Good laboratory procedure should be employed to avoid cross contamination of samples and reagents. Take out only the required volume of reagent from the original container for dispensing into wells. Discard unused reagents- do not return to containers

8. ASSAY PROCEDURE

Step 1: Reagents preparation

Allow the reagents to reach room temperature (18-30°C). Check the wash solution concentration for the presence of salt crystals. If crystals have formed in the solution, re-suspend by warming at 37°C until the crystals have dissolved. Dilute the stock wash fluid 20x times with distilled or deionised water. Use a clean vessel to dilute the solution. Prepare the Conjugate Solution.

Step 2: Numbering wells

Set the strips needed into the strip-holder and select sufficient number of wells to test one set of Standard controls (i.e. 9x wells), followed by 3x Negative controls, followed by required samples. Use only the number of strips required for the test (Use the separately provided DABA ELISA Frontsheet Template to record samples to be tested and procedural information).

Step 3: Adding controls and samples

Following a DABA ELISA Frontsheet Template as a guide, **pipette 90µl of Sample diluent and 10µl of each Standard Control** into their respective well positions (1A – 2A)**. Pipette 90µl of Sample diluent and 10µl of Negative Control** into their respective well positions (2B – 2D). **Pipette 98µl of Sample diluent and 2µl of each sample** into the remaining well positions (2E onwards) as designated on the DABA ELISA Frontsheet Template (only testing the number of samples in a single test run that can be dispensed into assigned wells within ten minutes).

Note: use a separate disposal pipette tip for each sample, negative control and standard control to avoid cross-contamination.

Step 4: Incubation

Cover the plate with a plate sealer and mix gently by tapping the side of the plate strip holder. Incubate **at 37 ± 2°C in a moist chamber or dry incubator for 60 ± 2 minutes** (If a dry incubator is used do not open the door frequently).

Step 5: Washing

After the end of the incubation, remove and discard the plate sealer. **Wash the wells five times with diluted wash buffer** (see reagent preparation). The wash cycle is carried out as follows: aspirate the contents of the well and dispense at least 300 µl per well of diluted wash buffer to form a meniscus, leave to soak for approximately 30-60 seconds and aspirate. Repeat the wash cycle a further four times. Alternatively an automatic plate washer may be used.

Step 6: Conjugate

After washing, tap the wells dry on absorbent paper. **Pipette 100µl of freshly prepared Conjugate Solution** into each well. This is best performed with a multichannel pipette. Cover the plate with a plate sealer and incubate **at 37 ± 2°C in a moist chamber or dry incubator for 120 ± 2 minutes** (If a dry incubator is used do not open the door frequently).

Step 7: Washing

After the end of the incubation, remove and discard the plate sealer. **Wash each well 5 times** as in Step 5.

Step 8: Substrate

After washing, tap the wells dry on absorbent paper. **Pipette 100µl of ready to use TMB Substrate** into each well. This is best performed with a multichannel pipette. Cover the plate with a plate sealer and incubate the plate **at 37 ± 2°C in a moist chamber or dry incubator for 30 minutes avoiding light**. The enzymatic reaction between the substrate and conjugate will produce a blue colour in the positive wells.

Step 9: Stopping reaction

Remove and discard the plate sealer. **Pipette 50µl of stop solution** into each well. This is best performed with a multichannel pipette. Mix gently by tapping the side of the plate strip holder. An intensive yellow colour will develop in the positive wells.

Step 10: Measure the absorbance

Read optical density at 450nm (if a dual filter instrument is used set the reference wavelength at 630 or between 620 and 650nm on the spectrophotometric plate reader). Note: read the absorbance within 10 minutes after stopping the reaction.

9. INTERPRETATION OF RESULTS AND QUALITY CONTROL

Each microplate must be considered separately when calculating and interpreting results of the assay, regardless of the number of plates concurrently processed. Evaluate the results ensuring the Optical Density (OD) values of the Negative Controls are within an acceptable range (see Quality Control below). Evaluate the 9 Standard Controls by entering the OD values of each Standard onto the electronic copy of the DABA Curve Excel Spreadsheet (provided separately) and running the ‘Solver’ Program. The quantified sample results are calculated by relating each sample OD value to the Standard Curve using the Solver Program on the DABA Curve Excel Spreadsheet

QUALITY CONTROL

The optical densities of all three Negative Controls should be < 0.1
Calculate the mean of all of the Negative Controls. If any Negative Control has an Optical Density > 0.1 this should be excluded from the mean calculation. Mean Optical Density of Negative Control should be <0.1.

Instructions for Running Solver Program on DABA Curve Excel Spreadsheet

Open windows Excel

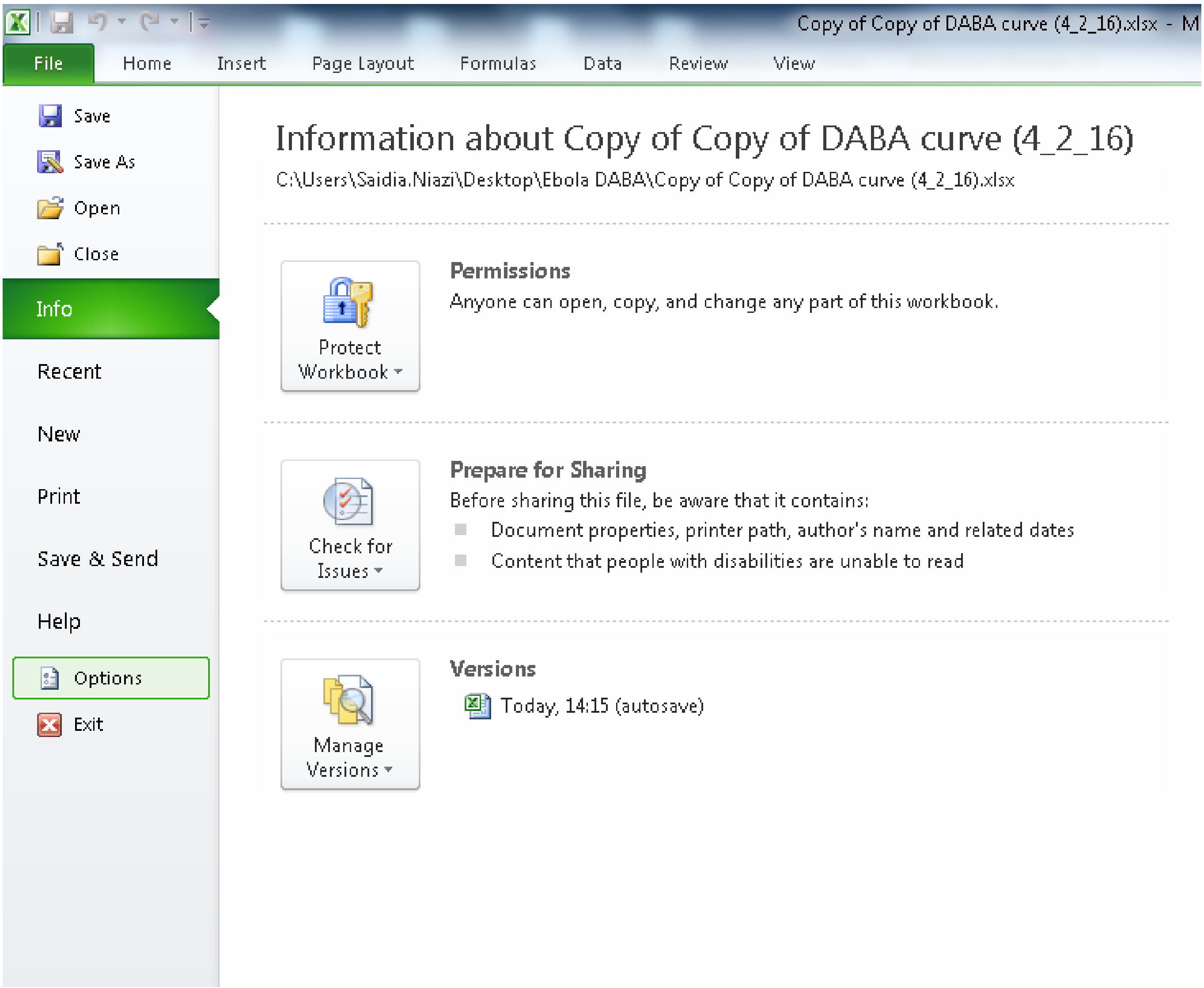

Click on FILE and select Options from the panel on the left hand side (as shown above)

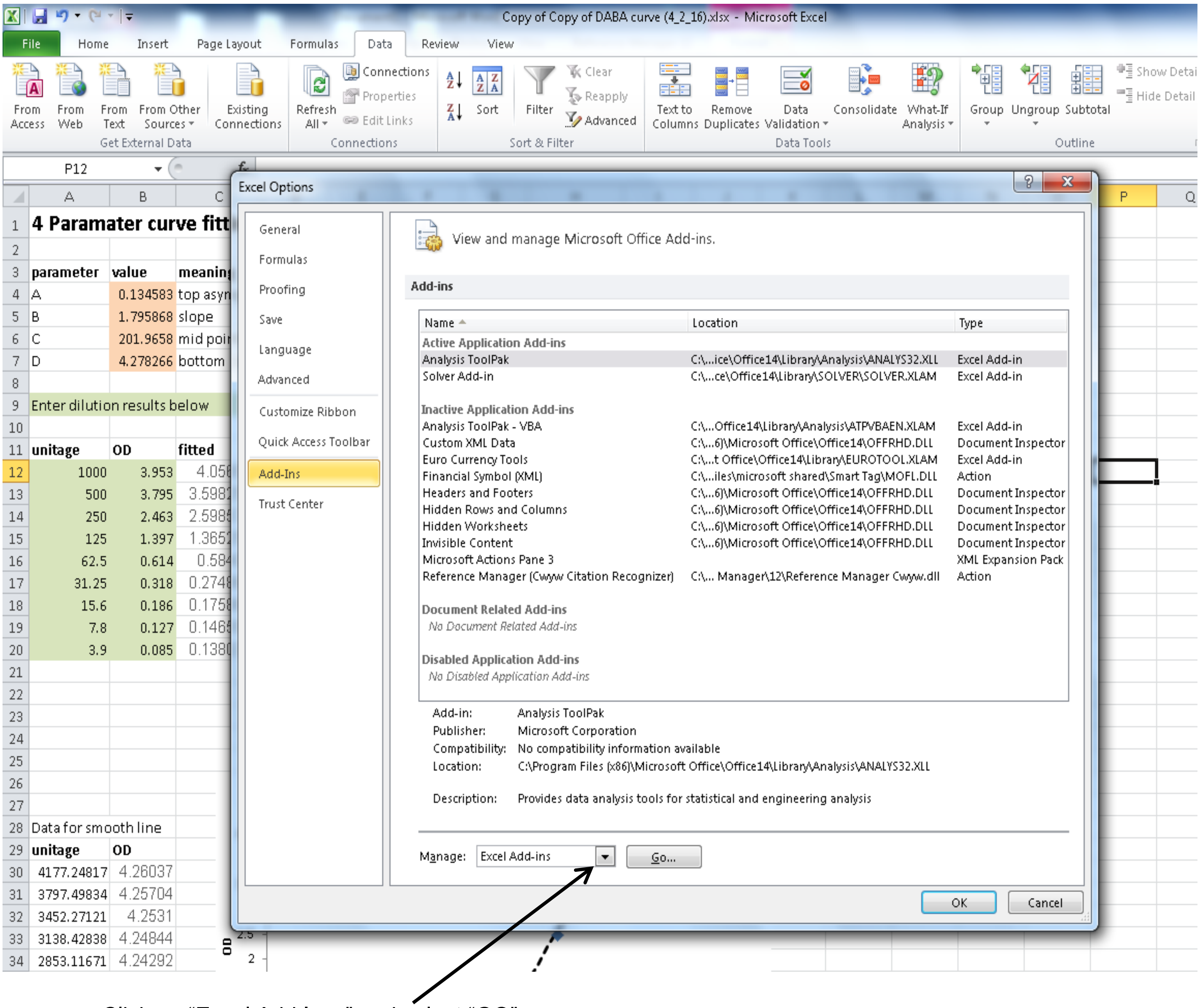

Click on “Excel Add Inns” and select “GO”

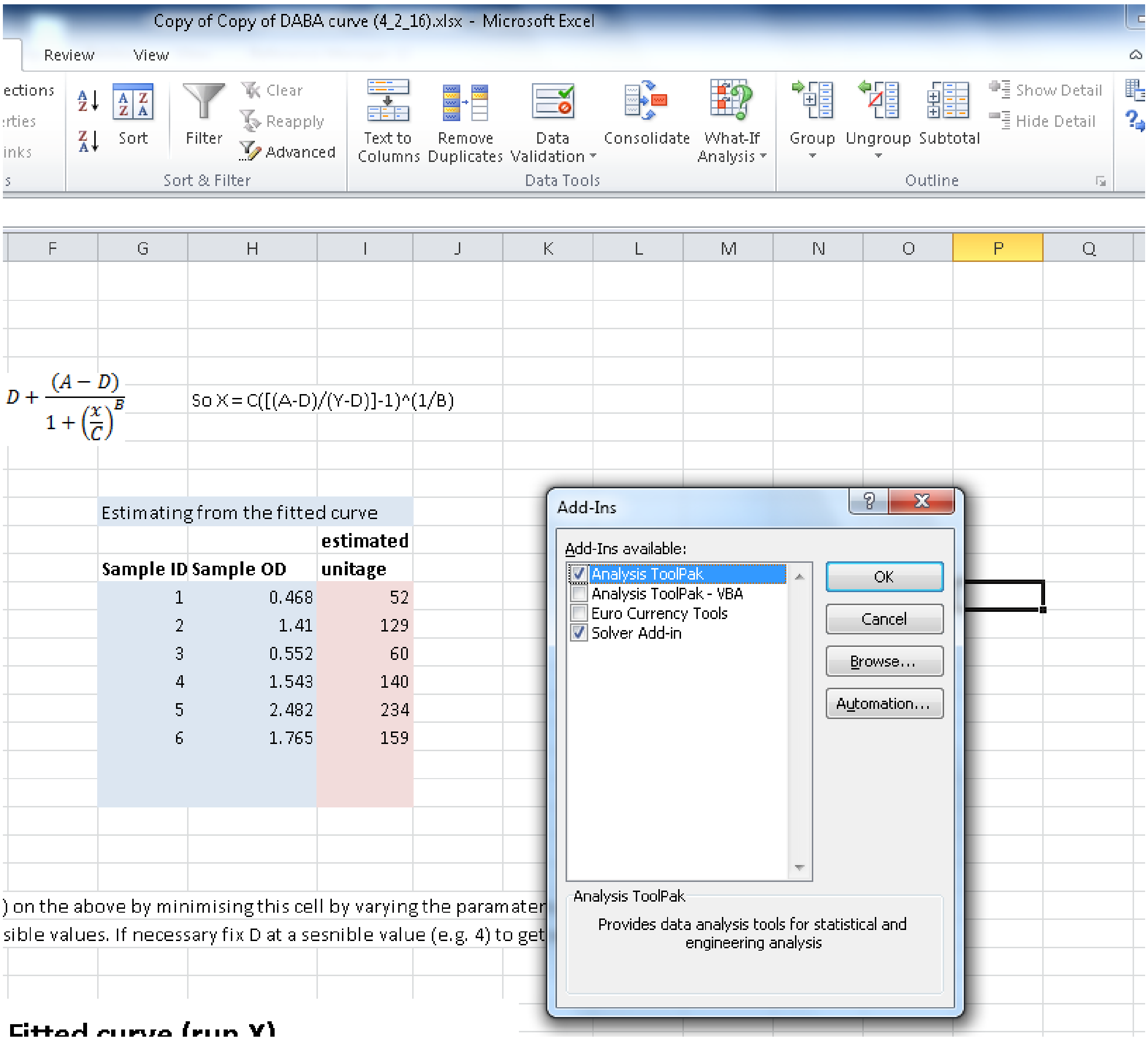

From the Add-Ins window select on “**Analysis Toolpack**” and “**Solver Add-Ins**”

Click OK

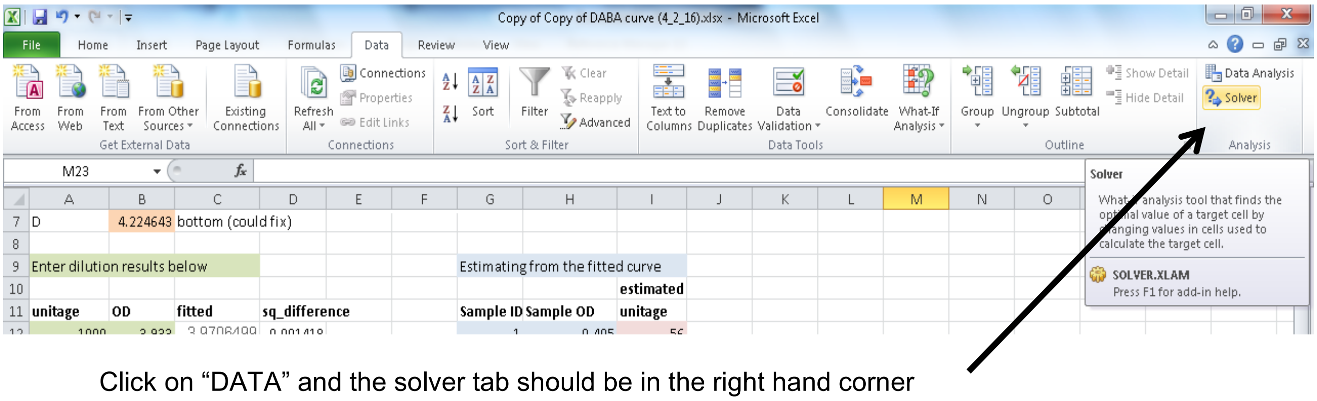

Click on “DATA” and the solver tab should be in the right hand corner

Using Solver to generate graph

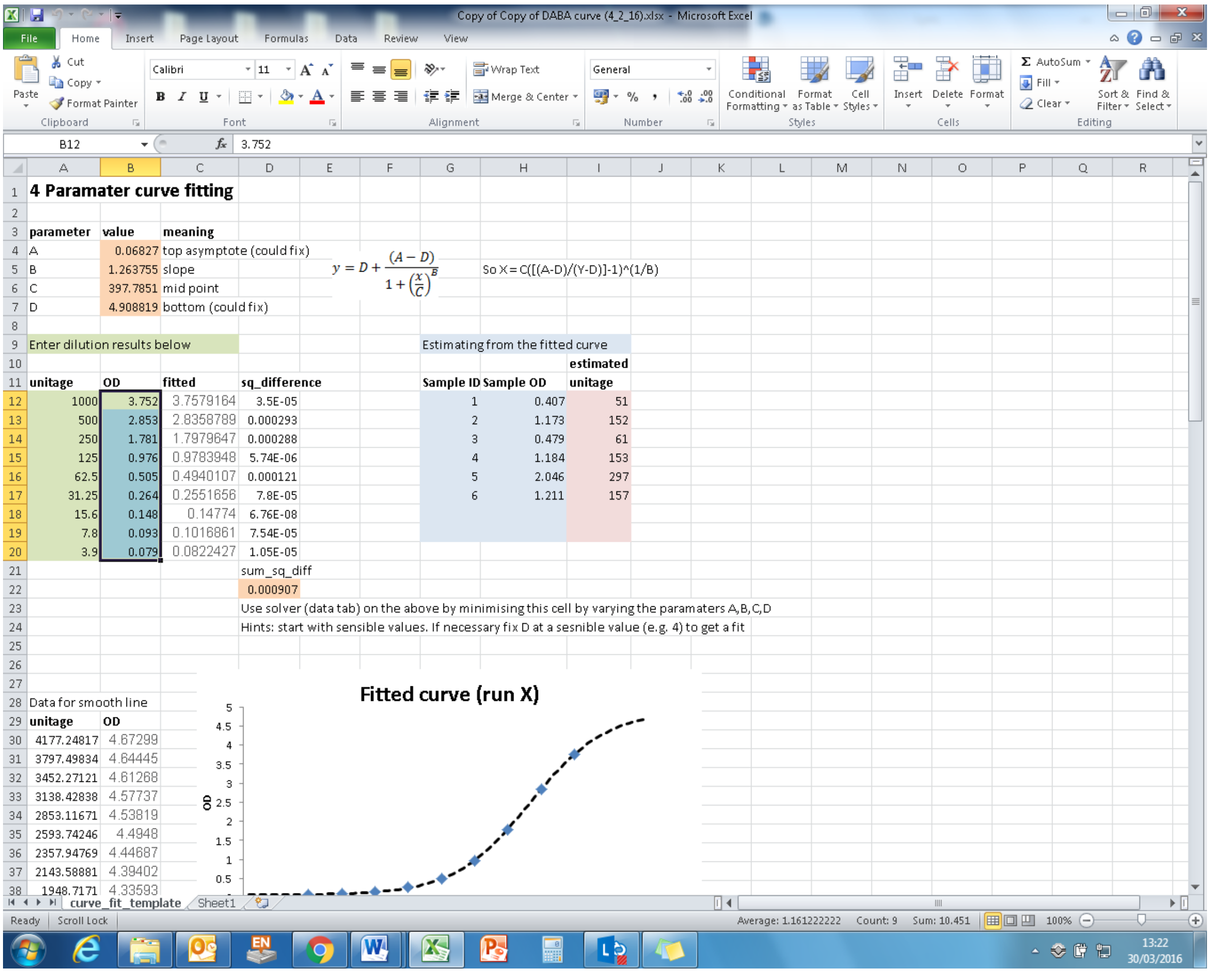

Copy the OD values for the 9 standards and paste in B12 to B20 as highlighted in the screenshot above

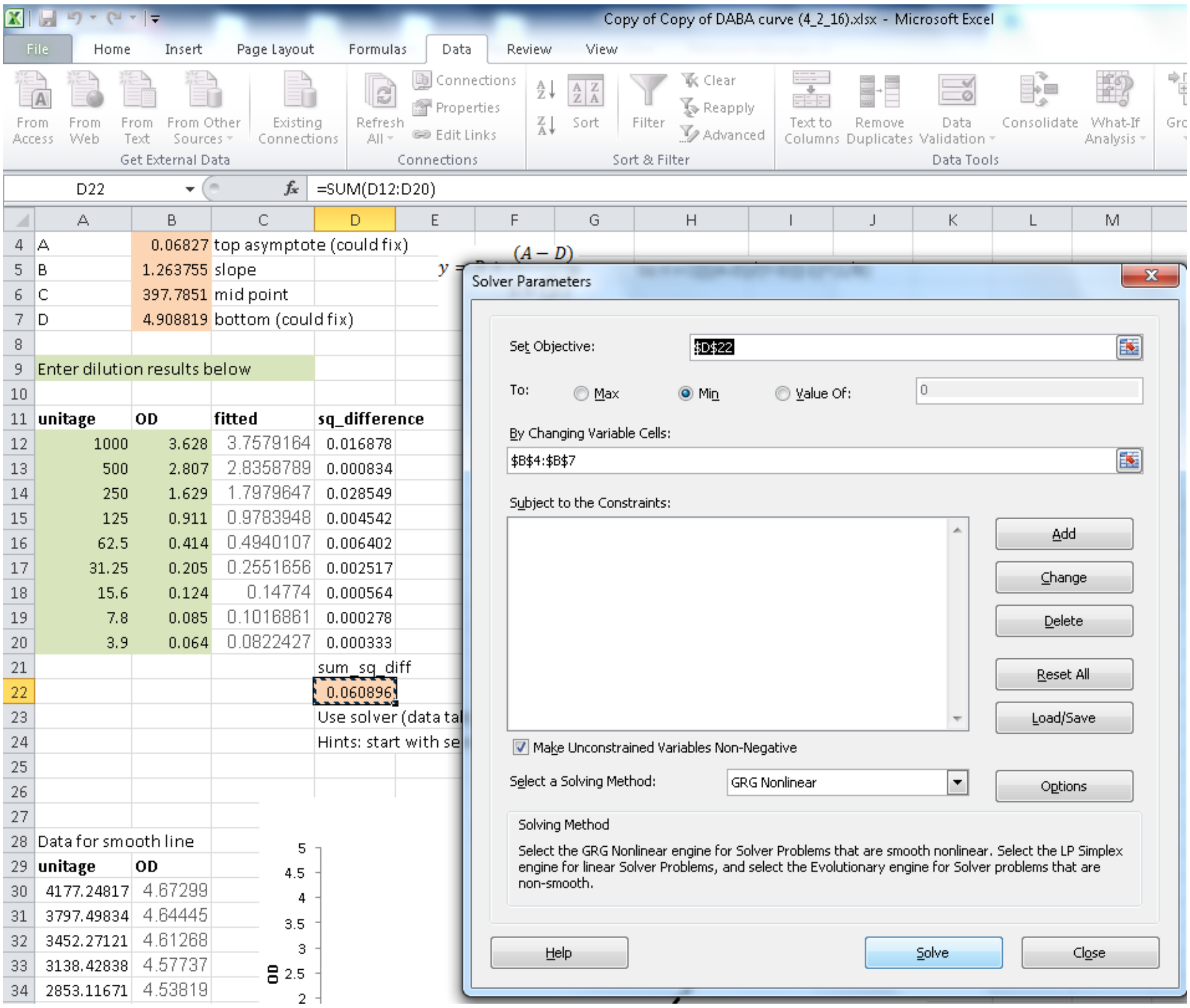

Click on DATA and then SOLVER

Ensure the Set Objectives has “D22” and click on Solve Icon button (highlighted ‘blue’ in the screen above)

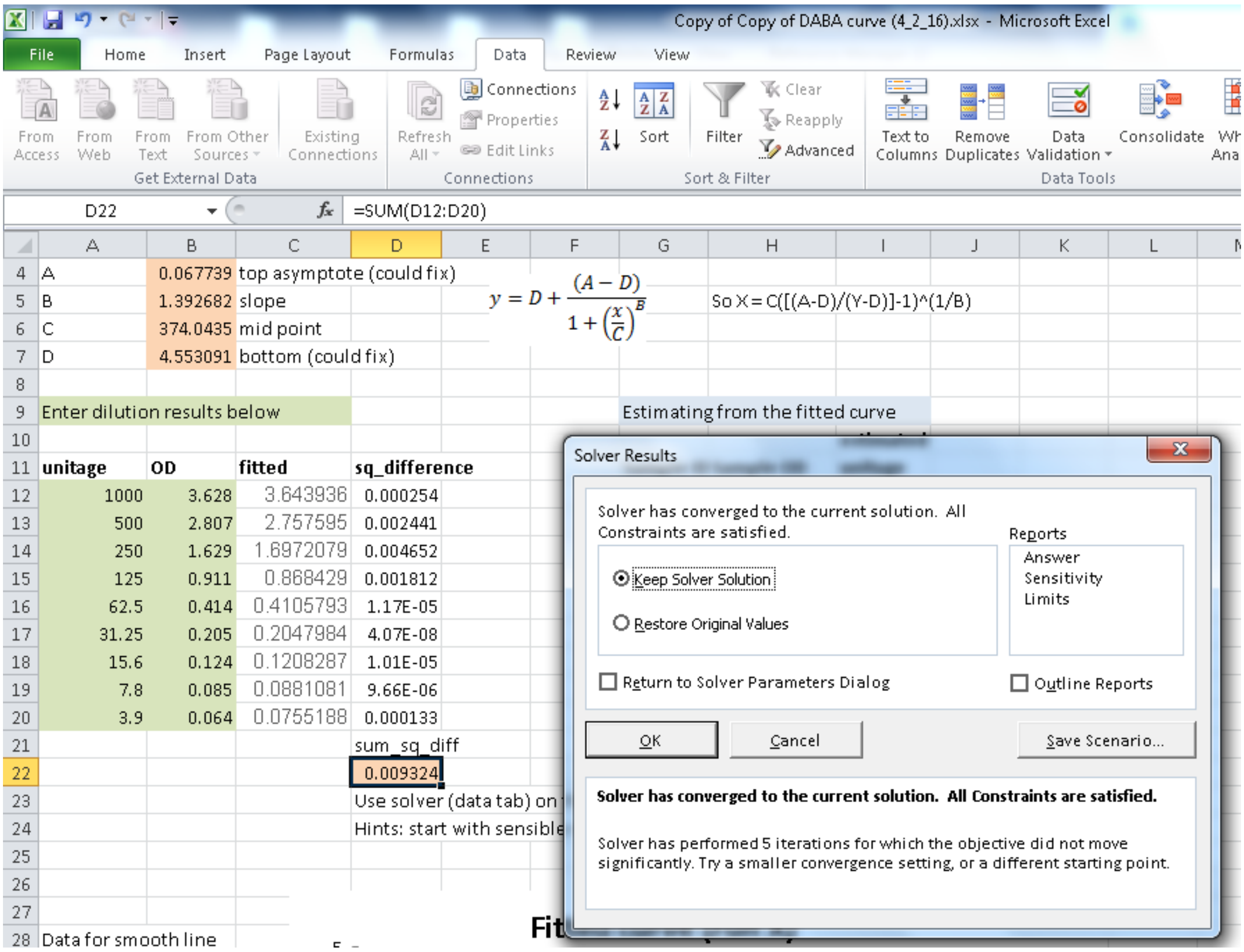

Click on keep Solver solution followed by OK

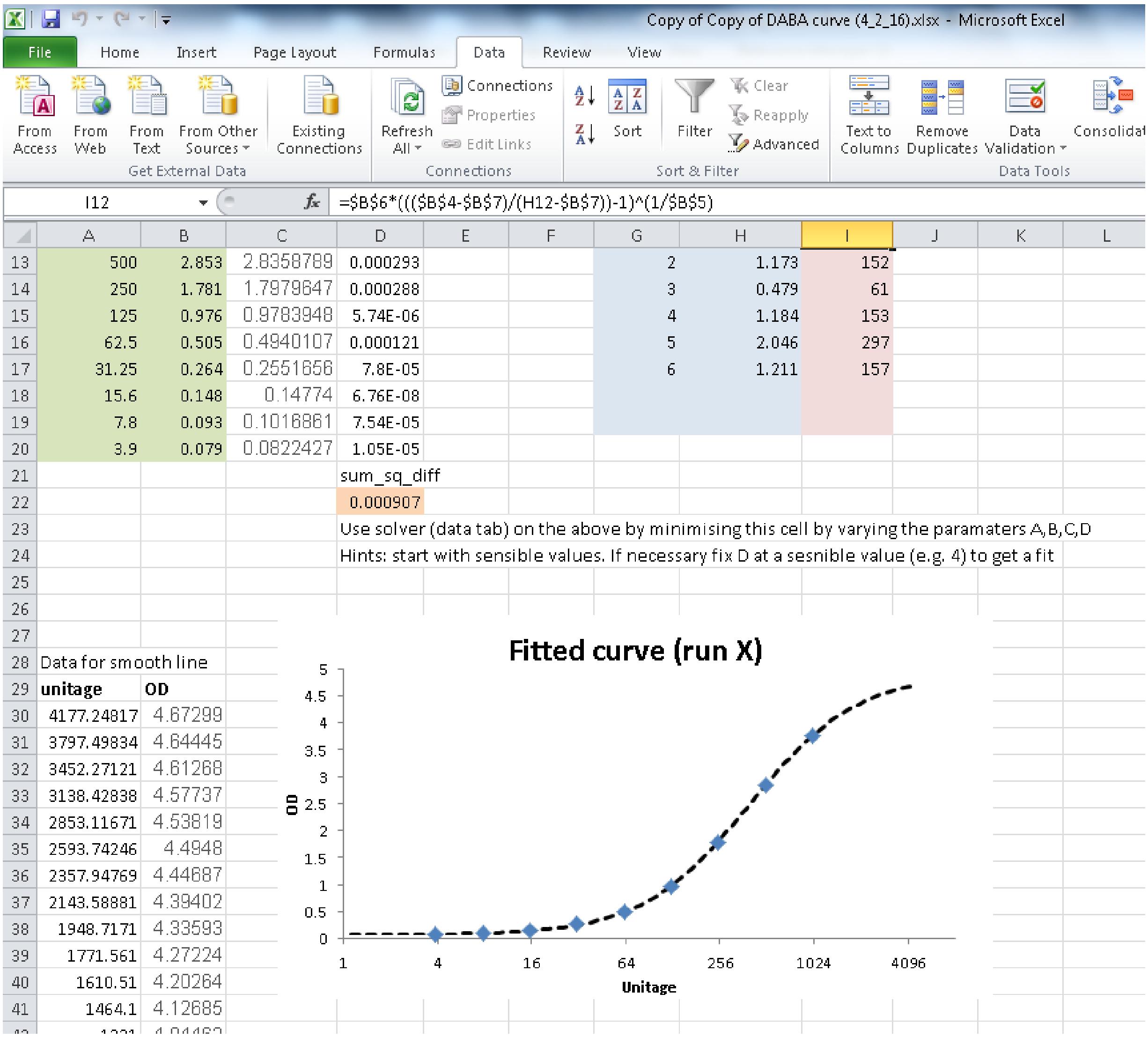

Standard points should now be fitted into the curve (the Lower the well **D22** value the closer the standard points are to the line)

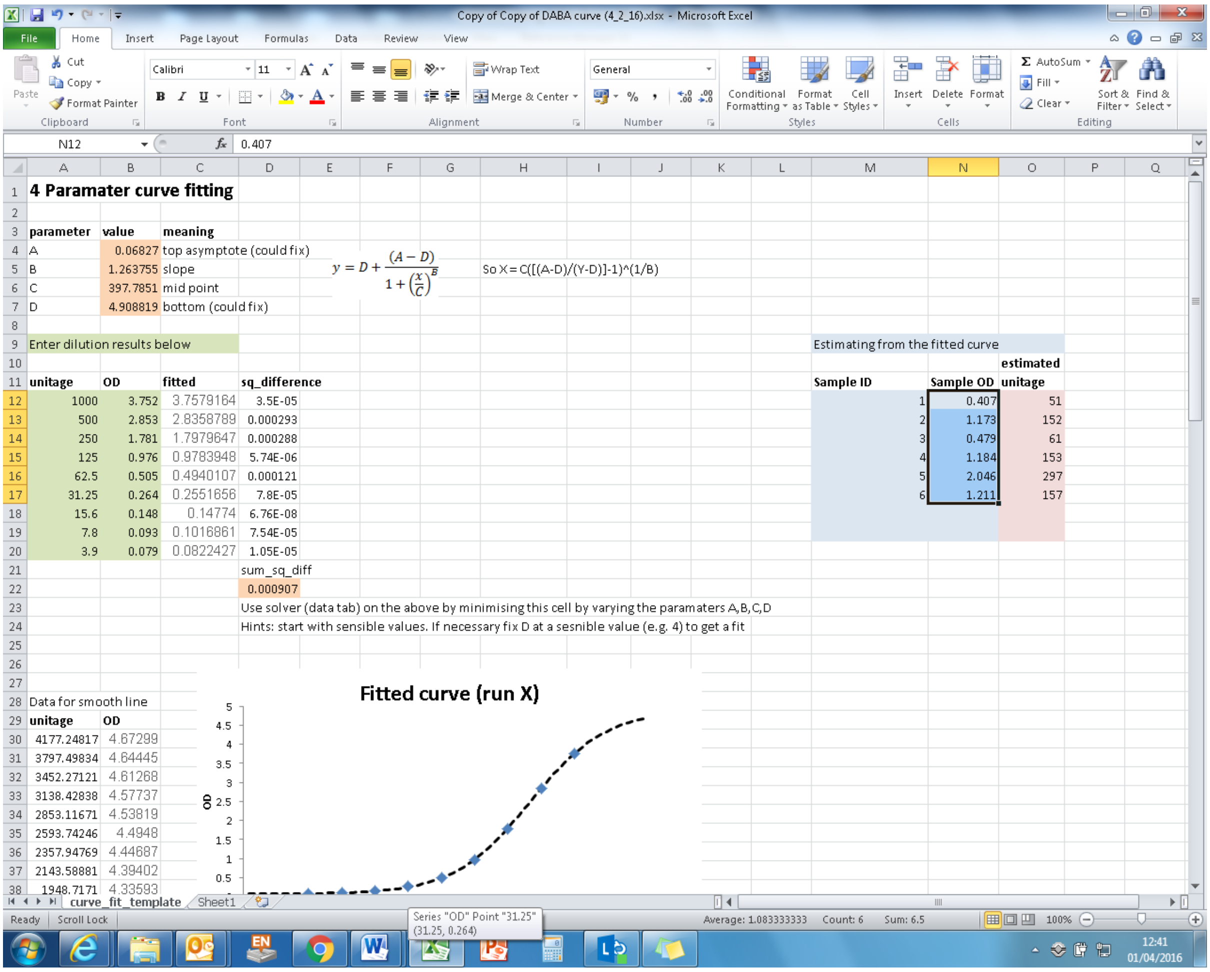

Copy the OD values of the samples and paste into Sample OD column N12 to N95 to calculate the 'Estimated Unitage' for each sample. Appropriate 'Sample Identifiers' can be typed into the 'Sample ID' column M12 to M95 as required.

As samples have been added to test wells at a 2µl input and Standards have been added at a 10µl input, the 'Estimated Unitage' is multiplied x5 to give an 'Assigned Unitage' in Arbitrary units per ml. This is performed automatically on the DABA Curve Excel Spreadsheet provided.

INTERPRETATION OF RESULTS

Samples with an Optical Density too low to calculate an 'Estimated Unitage' from the fitted curve i.e Estimated Unitage listed as '#NUM!' after running 'Solver' Program should be resulted as containing <100 Arbitrary units per ml.

The Assigned Unitage is only an Arbitrary value derived from the Standard curve. The Assigned Unitage value is a direct correlation of the Antibody Titre of EBOV antibodies in the Sample. Samples with a greater Assigned Unitage value are likely to contain higher titres of antibodies to EBOV GP.

EBOLA DABA Qualitative Assay

Positive Control = 2x rabbit anti-ZEBOV VLP antisera diluted 1:1K in NHP (position 1A-1B)

Negative Control = 4x Negative Human Plasma

Conjugate = rZEBOV GP-HRP (batch 29/04/15 used at 1:8K dilution in Murex Conjugate Diluent)

Procedure:

- Add 100µl of sample and control
- Incubate for 1 hour at 37°C
- Wash 5x in MUREX Glycine Borate wash buffer
- Add 100µl of conjugate
- Incubate for 2 hours at 37°C
- Wash 5x in MUREX Glycine Borate wash buffer
- Add 100µl TMB Ready to Use Substrate
- Incubate for 30 mins at 37°C
- Stop with 50µl 2M H_2SO_4 and read at 450/630 nm

Competitive enzyme-linked immunosorbent assay for the detection of ebola virus antibody in human serum or plasma

1. SUMMARY

EBOV Comp is developed to detect anti-EBOV antibodies to Zaire ebolavirus.
EBOV Comp has been developed and designed to be extremely sensitive and specific using recombinant proteins: recombinant ZEBOV GP (IBT bioservices) and Anti-EBOV Monoclonal AB-HRP.

2. INTENDED USE

This kit is an enzyme-linked immunosorbent assay for the qualitative detection of antibodies to Zaire ebolavirus in human serum or plasma. It is intended as a confirmatory assay for individuals who were screen reactive using the EBOV G-capture assay.

3. PRINCIPLE OF THE TEST

The EBOV Comp ELISA is based on one step incubation competitive principle ELISA. Anti-EBOV antibodies if present in the sample, compete with monoclonal anti-EBOV antibodies, conjugated to horseradish peroxide (HRP) for a fixed amount of recombinant ZEBOV GP pre-coated in the wells. When no anti-EBOV antibodies is present in the sample, the HRP labelled anti-EBOV will be bound with the ZEBOV GP antigens and after adding TMB substrate the colourless chromogens are hydrolysed by the bound HRP conjugate to a blue coloured product. The blue colour turns yellow after stopping the reaction with 2M sulphuric acid. No or low colour developing suggests the presence of antibodies to EBOV in the sample.

4. REAGENTS

Each kit contains sufficient materials for 96 tests. The shelf life of each kit is as indicated on the label fixed to the box containing the kit.

Materials Provided:

**Coated Wells**:	1 plate of 96 wells coated with recombinant ZEBOV GP. Allow the wells to reach room temperature (18 to 30°C) before removal from the bag. Place unused wells in the sealable storage bag provided and return to 2 to 8°C.
**Negative Control**:	One bottle containing 500 µl of normal human plasma
**Positive Control**:	One bottle containing 250 µl of positive inhibition control
**Conjugate Diluent:**	One bottle containing 10 ml of buffered diluent
**Conjugate Concentrate:**	One bottle containing 500 µl of 35 times strength monoclonal anti-EBOV coupled with horseradish peroxidase.<br><br>Add one volume of Conjugate Concentrate to 34 volumes of Conjugate Diluent to get the required volume.
**TMB Substrate - Ready to Use**:<br>**(supplied by MicroImmune Ltd)**	One bottle containing 10 ml of 3,3’,5,5’- tetramethylbenzidine and stabilisers in a colourless solution (TMB Substrate – ready to use)<br><br>Keep substrate away from sunlight. The Substrate Solution should be colourless; if it is purple before being used, it should be discarded and fresh Substrate Solution used.<br><br>Once opened, the bottle of TMB Substrate is stable refrigerated (2 to 8°C) for 30 days, but must be discarded if crystals have formed.
**Stop Solution (2M H_2SO_4)**:	One bottle containing 5 ml of 2M sulphuric acid (H_2SO_4) ready to use.
**Wash Fluid**:	One bottle containing 125 ml of 20 times working strength of phosphate buffer saline, Tween 20 and preservative.<br><br>Add one volume of Wash Fluid Concentrate to 19 volumes of distilled or deionised water to give the required volume or dilute the entire contents of one bottle of Wash Fluid to a final volume of 2500 ml.

Additional Material and Instruments Required But Not Provided:

Good quality deionised or distilled water
Clean vessels for wash buffer preparation

Microtitre plate cover
Micropipettes and disposable tips capable of delivering 1000 µL, 100 µL and 1-5µl volumes.
Waste discard container with disinfectant
EIA plate reader capable of reading optical density at 450nm (and 620-650nm).
Incubator, 37°C

5. SPECIMEN COLLECTION AND PREPARTION

Either fresh serum or plasma samples can be used for this assay. If not used immediately, they can be stored at 2-8°C for one week. Care should be taken to ensure that the serum samples are clear and not contaminated by microorganisms. Plasma samples collected into EDTA, sodium citrate or heparin may be tested, but highly lipaemic, icteric or haemolysed samples should be used as they can give false results in the assay. Do not heat inactive samples.

6. STORAGE AND STABILITY

When stored at 2-8°C, the kit is stable up to the expiration date printed on the kit label.

7. PRECAUTIONS AND SAFETY

The Positive Control serum and Negative Control serum are not reactive for antibodies to HIV 1 and 2, HCV and for Hepatitis B surface antigen. The controls should be handled and disposed of as though potentially infectious.

The TMB substrate solution contains 3, 3', 5, 5'-tetramethylbenzidine and has been reported to be non-carcinogenic. Avoid direct skin- contact with the reagent. Wear latex gloves when handling this reagent. If TMB comes into contact with the skin, wash immediately with water.

The Stop Solution contains sulphuric acid (2M). Contact with skin and mucous membranes should be avoided. If the Stop Solution comes into contact with these sites, rinse with copious amounts of water.

Wear disposable gloves when handling clinical specimens and kit components. Treat all clinical specimens and controls and any materials coming into contact with them as potentially infectious.

Dispose clinical material and potentially infected materials in accordance with local regulations.

Avoid microbial contamination of reagents. Do not use reagents that show signs of contamination.

Good laboratory procedure should be employed to avoid cross contamination of samples and reagents. Take out only the required volume of reagent from the original container (usually 0.9- 1.0 mL per strip) for dispensing into wells. Discard unused reagents- do not return to containers

8. ASSAY PROCEDURE

Step 1: Reagents preparation

Allow the reagents to reach room temperature (18-25°C). Check the wash buffer concentration for the presence of salt crystals. If crystals have formed in the solution, re-suspend by warming at 37°C until the crystals have dissolved. Dilute the stock washer buffer 20x times with distilled or deionised water. Use a clean vessel to dilute the buffer. Prepare the Conjugate Solution.

Step 2: Numbering wells

Set the strips needed in strip-holder and number sufficient wells including 2 positive and 4 negative controls. Use only the number of strips required for the test.

Step 3: Adding sample and conjugate

Add **25µl of positive control, negative control and sample** into their respective wells. Note: use a separate disposal pipette tip for each sample, negative control and positive control to avoid cross-contamination. Add **75µl of conjugate** to each well and mix gently by tapping the plate.

Step 4: Incubation

Cover the plate with a plate sealer and incubate for **60 minutes at 37°C**. If a dry incubator is used do not open the door frequently.

Step 5: Washing

After the end of the incubation, remove and discard the plate sealer. Wash each well **5 times** with diluted wash buffer by aspirating the contents of the well and dispense at least 300 µl per well of wash buffer to form a meniscus. Each time allow the microwells to soak for 30-60 sec. After the final washing cycle turn down the strips plate onto blotting paper or clean towel and tap the plate to remove any remaining wash buffer.

Step 6: Substrate

Dispense **100µl of ready to use TMB substrate** into each well. Cover the plate with a plate sealer and mix gently by tapping the plate. Incubate the plate at **37°C for 30 minutes** avoiding light. The enzymatic reaction between the substrate and conjugate will produce a blue colour in the negative control and anti-EBOV negative sample wells.

Step 7: Stopping reaction

Remove and discard the plate sealer. Add **50µl of stop solution** into each well and mix gently. Intensive yellow colour develops in the negative control and anti-EBOV negative sample wells.

Step 8: Measure the absorbance

Calibrate the plate reader with the blank well and read the absorbance at 450nm. If a dual filter instrument is used set the reference wavelength at 630nm. Calculate the cut-off value and evaluate the results. Note: read the absorbance within 10 minutes after stopping the reaction.

9. INTERPRETATION OF RESULTS AND QUALITY CONTROL

Each microplate must be considered separately when calculating and interpreting results of the assay, regardless of the number of plates concurrently processed. The results are calculated by relating each sample optical density (OD) value to the cut-off value (CO) of the plate.

Calculation of cut-off value:

Cut-off value (CO) = NC_{mean} + PC_{mean} / 2

NC_{mean} = the mean absorbance value for 4 negative controls

If one of the negative control values does not meet the Quality Control range specifications, it should be discarded and the mean value calculated again using the remaining values.

PC_{mean} = the mean absorbance value for the positive controls

Quality Control Range

The test results are valid if the Quality Control criteria are verified:

The absorbance value OD of the negative control must be greater than 0.800

The absorbance value OD of the positive control must be less than 0.100

<u>Interpretation of results</u>

Positive results (S/CO ≤1): Samples giving absorbance less or equal to the cut-off value are positive for this assay, that is, antibodies to EBOV have been detected with this ELISA kit.

Negative results (S/CO >1): Samples giving an absorbance greater than the cut-off value are considered negative, that is no antibodies to EBOV have been detected using this ELISA kit.

Immunoglobulin G capture enzyme immunoassay for the detection of antibodies to Ebola virus in human serum, plasma or oral fluid

1. INTENDED USE

EBOV G CAPTURE is an enzyme immunoassay for the detection of antibodies to Ebola virus (EBOV) in human serum, plasma or oral fluid. It may be adapted for use with other analytes such as lysed blood or eluates from dried blood spots, advice should be sought. This assay is to be performed for validation purposes only.

2. SUMMARY

EBOV G CAPTURE has been developed to be used to monitor prevalence of EBOV specific IgG antibodies to EBOV glycoprotein (GP) in population studies. Additionally, the assay can be used on oral fluids to confirm that a "recovered" individual offering their blood as a convalescent donor has detectable high level antibody to EBOV GP in their blood stream.

3. REAGENTS

Each kit contains sufficient materials for 96 tests. The shelf life of each kit is as indicated on the label fixed to the box containing the kit. All components must be stored at 2 to 8°C unless otherwise stated.

**Coated Wells**:	1 plate of 96 wells coated with anti-human IgG antibody. Allow the wells to reach room temperature (18 to 30°C) before removal from the bag. Place unused wells in the sealable storage bag provided and return to 2 to 8°C.
**Transport Medium**:	One bottle containing 100 ml of phosphate buffered saline, protein stabiliser and detergent.
**Negative Control**:	One bottle containing 2 ml of negative control material. Ready to use.
**Positive Control**:	One bottle containing 1 ml of positive control material. Ready to use.
**Conjugate Diluent:**	One bottle containing 10 ml of buffered diluent.
**Conjugate Concentrate:**	One bottle containing 100 µl of 100 times strength recombinant EBOV Zaire glycoprotein coupled with horseradish peroxidase antigen. **NB: Vortex/Centrifuge tube to ensure the full volume of Conjugate Concentrate is in bottom of tube prior to opening.**<br><br>Add one volume of Conjugate Concentrate to 99 volumes of Conjugate Diluent to get the required volume.
**TMB Substrate - Ready to Use**:<br>**(supplied by MicroImmune Ltd)**	One bottle containing 10 ml of 3,3',5,5'- tetramethylbenzidine and stabilisers in a colourless solution (TMB Substrate – ready to use)<br><br>Keep substrate away from sunlight. The Substrate Solution should be colourless; if it is purple before being used, it should be discarded and fresh Substrate Solution used.<br><br>Once opened, the bottle of TMB Substrate is stable refrigerated (2 to 8°C) for 30 days, but must be discarded if crystals have formed.
**Stop Solution (2M H_2SO_4)**:	One bottle containing 5 ml of 2M sulphuric acid (H_2SO_4) ready to use.
**Wash Fluid**:	One bottle containing 125 ml of 20 times working strength of Glycine Borate Wash Solution.<br><br>Add one volume of Wash Fluid Concentrate to 19 volumes of distilled or deionised water to give the required volume or dilute the entire contents of one bottle of Wash Fluid to a final volume of 2500 ml.<br><br>Store the working strength Wash Fluid at 18 to 30°C in a closed vessel under which conditions it will retain activity for one month.
**Oral Fluid Collection Device (Oracol):**	Follow instructions to use. Can be stored at 18 to 30°C

MATERIALS REQUIRED BUT NOT PROVIDED

• Good quality deionised or distilled water.

• Tubes suitable for diluting serum specimens and microtitre plate cover.
• Micropipettes and disposable tips capable of delivering 1000 µL, 100 µL and 1-5µl volumes.
• Waste discard container with disinfectant
• EIA plate reader capable of reading optical density at 450nm (and 620-650nm).
• Incubator, 37°C
• Washer or Aspirator

REAGENT PREPARATION

Warm the wash buffer to re-dissolve any salts that may have formed on storage. Prepare working strength wash buffer by adding 1 part wash fluid concentrate to 19 parts of distilled or deionised water. It is recommended that working strength buffer be prepared as required on the day of use. Remaining wash buffer concentrate should be stored at 2-8°C. Sufficient wash buffer is provided to enable five washes of each well at each stage of the procedure.

To prepare the Conjugate Solution add one volume of Conjugate Concentrate to 99 volumes of Conjugate Diluent to get the required volume.

All other reagents are provided ready-to-use.

Bring all reagents to ambient room temperature prior to use.

WARNINGS AND PRECAUTIONS

• The Positive Control and Negative Control are not reactive for antibodies to HIV 1 and 2, HCV and for Hepatitis B surface antigen. The Positive Control has been prepared from a plasma donation known to have come from a convalescent individual who was repeatedly nucleic acid free at the time of donation on both their oral fluid and plasma.

• The TMB substrate solution contains 3, 3', 5, 5'-tetramethylbenzidine and has been reported to be non-carcinogenic. Avoid direct skin- contact with the reagent. Wear latex gloves when handling this reagent. If TMB comes into contact with the skin, wash immediately with water.

• The Stop Solution contains sulphuric acid (2M). Contact with skin and mucous membranes should be avoided. If the Stop Solution comes into contact with these sites, rinse with copious amounts of water.

• Wear disposable gloves when handling clinical specimens and kit components. Treat all clinical specimens and controls and any materials coming into contact with them as potentially infectious.

• Dispose of clinical material and potentially infected materials in accordance with local regulations.

• Avoid microbial contamination of reagents. Do not use reagents that show signs of contamination.

• Good laboratory procedure should be employed to avoid cross contamination of samples and reagents. Take out only the required volume of reagent from the original container for dispensing into wells. Discard unused reagents, do not return to containers.

4. SPECIMEN COLLECTION

Handle all oral fluid, blood, serum and plasma as potentially infectious material.

Serum and plasma (EDTA, citrated or heparinised) samples are suitable specimens for the test and should be obtained from whole blood using standard laboratory procedure.

Oral fluid specimens should be collected as described on the swab packaging for the oral fluid collection devices. Prior to performing the EBOV G CAPTURE assay the saliva should be extracted from the swabs as follows:

- Add 1 ml of Transport Medium to tube containing swab.
- Vortex or agitate swab manually for 20 seconds to ensure foaming of transport medium.
- Remove swab from tube using a twisting motion to extract as much liquid as possible from the swab.
- Carefully discard the swab.
- Extracted saliva can now be recovered from the tube.
- Store at 2 to 8°C prior to testing

5. PROCEDURE

I. Bring all reagents to room temperature (18-25°C) before use. This is very IMPORTANT.

2. Dilute the serum or plasma samples 1:200 in Transport Medium by dispensing 2 µl of sample into a labelled tube and adding 400 µl of Transport Medium and mix. Oral fluid samples should not be diluted.

3. Remove and assemble the required number of anti-human IgG microwell strips to perform the test. A minimum of 6 wells is needed for the controls which must be included in each test run. Return unused microwell strips and the desiccant to the foil pouch and reseal.

4. Pipette I00 µl per well of the positive and negative controls to assigned wells, two wells for the positive control and four wells for the negative control. The positive and negative controls do not need diluting, they are ready to use.

5. Pipette I00 µl per well of the oral fluid or diluted serum or plasma samples to assigned wells. Only test the number of samples, in a single test run, that can be dispensed into assigned wells within ten minutes. Cover microtitre plate with lid or sealing tape.

6. Incubate at 37 ± 2°C in a moist chamber for 60 ± 2 minutes.

7. Wash wells five times with diluted wash buffer (see reagent preparation). The wash cycle is carried out as follows: aspirate the contents of the well and dispense at least 300 µl per well of diluted wash buffer to form a meniscus, leave to soak for approximately 30 seconds and aspirate. Repeat the wash cycle four further times. Alternatively an automatic plate washer may be used.

8. After washing, tap the wells dry on absorbent paper. Pipette 100 µl per well of Conjugate Solution to the wells, cover plate and incubate at 37 ± 2°C in a moist chamber for 120 ± 2 minutes.

9. Wash the well five times with wash buffer as in step 7 and tap the wells dry on absorbent paper.

10. Pipette 100 µl per well of ready to use TMB Substrate. This is best performed with a multichannel pipette.

11. Incubate for 30 ± 2minutes at 37 ± 2°C protected from strong light.

I2. Pipette 50 µl per well of the Stop Solution. The stop solution should be added using the same timing and sequence used to add the substrate solution.

I3. Read optical density at 450nm (set the reference wavelength at 630 or between 620 and 650nm, if available on the spectrophotometric plate reader).

6. RESULTS

QUALITY CONTROL

The optical density of the Positive Control should be > 1.0
The optical density of the Negative Control should be < 0.1
Calculate the mean of all of the Negative Controls. If any Negative Control has an optical density > 0.1 this should be excluded from the mean calculation.

CUT-OFF VALUE

Calculate the Cut-off Value by adding 0.1 to the mean of the Negative Controls.

INTERPRETATION OF RESULTS

Non-Reactive Results: Samples giving an absorbance less than the Cut-off Value are considered non-reactive.
Reactive Results: Samples giving an absorbance equal to or greater than the Cut-off Value are considered reactive in this assay.

Measurement of neutralising antibody.

Single-cycle infectious EBOV GP pseudo-type retrovirus was produced in HEK-293 cells (1) by co-transfection of a replication-defective HIV plasmid (pSG3$^{\Delta env}$, Tranzyme Inc., Durham, NC) and EBOV-GP plasmid EBOV 2012/2014 GP sequence derived from KP096421 (carrying later appearing mutations A81V, I371V , T229A and N551D; GeneArt/ThermoFisher) using Lipofectamine 3000 (Invitrogen) as previously described (2). Vesicular stomatitis glycoprotein (pHEF-VSVG, NIH AIDS Reagent Program) was utilised as a positive control for transfection efficiency and pSG3$^{\Delta env}$ alone as a negative control. The culture supernatant containing pseudo-type virus was harvested after 48 hours of culture and stored at −80°C prior to use. HEK-293 and TZM-bl cell (NIH AIDS Reagent Program) culture, virus infectivity and plasma neutralizing capacity were as previously described (2). In brief TZM-bl (3) cells in a 96-well plate format were cultured for two days following inoculation with serial dilutions of convalescent plasma and a fixed input of pseudo-type virus. The cells were washed, lysed in reporter lysis buffer and luciferase activity measured using a Luciferase Assay kit (Promega, Madison, WI). All infections were performed in triplicate and repeated. Cells inoculated with control glycoprotein-deficient pseudo-type virus were used to determine background luciferase activity. Non-linear regression curves were determined and IC_{50} values calculated using Inhibitor versus Response variant of the Non Linear Regression function of GraphPad Prism version 5.00 for Windows, (GraphPad Software, San Diego California USA, www.graphpad.com). A significance level of P <0.05 was used for all analyses. Inhibition was normalised to virus infectivity in the absence of convalescent plasma and an IC_{50} was determined as the interpolated plasma dilution which would give 50% inhibition of the luciferase signal.

1. Wei X, Decker JM, Wang S, et al. Antibody neutralization and escape by HIV-1. Nature **2003**; 422(6929):307-12.

2. Baan E, de Ronde A, Stax M, et al. HIV-1 autologous antibody neutralization associates with mother to child transmission. *PloS one* **2013**; **8**(7): e69274

3. Wei X, Decker JM, Liu H, et al. Emergence of resistant human immunodeficiency virus type 1 in patients receiving fusion inhibitor (T-20) monotherapy. Antimicrobial agents and chemotherapy 2002; 46(6): 1896-905.
